# Assessments of empathy in medical school admissions: what additional evidence is needed?

**DOI:** 10.5116/ijme.52b7.5294

**Published:** 2014-01-11

**Authors:** Mohammadreza Hojat

**Affiliations:** Center for Research in Medical Education and Health Care, Department of Psychiatry and Human Behavior, Jefferson Medical College of Thomas Jefferson University, USA

## Introduction

The Association for the Study of Medical Education (ASME) sponsored a symposium on the theme of Examining the Evidence with Regard to Character, Personality and Values in Medical School Selection which was held on October 14, 2013 at the University of Sheffield Medical School in the United Kingdom. I was invited to speak about credibility issues related to personality assessments in health profession educations. To my pleasant surprise, I found the European audience receptive (more than their counterparts in the United States) to the idea of using personality assessments in admission decisions. There seems to be a hesitation among leaders in medical education in the United States to use personality assessments for selection purposes. They argue that convincing evidence is needed to support using personality assessments in medical school admission. In my presentation, I provided evidence to refute the argument against using personality assessments in admission decisions. Because of our extensive research at Jefferson Medical College on the topic of empathy in medical education and patient care, I placed the emphasis on credibility of evidence for using assessments of empathy, as a personality attribute, in the selection of applicants and professional development of students in any academic health profession institution. The editor of this journal who has a keen interest in medical education issues attended the symposium and suggested that I write an opinion piece about the issue for international audience of the journal. This editorial is based, in part, on my presentation at that symposium.

### Why is personality relevant to medical school admissions?

There is a consensus among behavioral and social scholars that personality plays an unquestionable role in human behavior. In the practice of medicine, the importance of personality in professional assessments has been acknowledged in a paradigm of physician performance.^1^^,^^2^ There is a large volume of research in medical education about the contribution of personality to academic achievement, clinical competence, and specialty interest of doctors-in-training and in-practice.^3^ The notion that personality is a contributing factor to academic achievement, clinical competence, career choice, and professional behavior implies that personality should be considered as a pertinent measure not only for the assessment of professional development of doctors-in-training, but more importantly as an additional requirement for the admission of qualified applicants to medical schools.

### Which personality attributes would be more credible?

A vast array of personality measures has been used in medical education research.^3^ The crucial question is which personality attributes are more credible for the assessments of professional development of doctors-in-training, and for consideration in the admission of applicants to medical schools? The choice of pertinent personality attributes should be based on the following three requirements:

1. Conceptual relevancy: the personality attributes of choice must be conceptually relevant to clinical competence and optimal patient outcome which is the ultimate goal of medical education.^4^ Obviously, a lack of clear conceptual relevancy between selected personality attributes and indicators of clinical competence and patient outcomes would not only undermine the potential value of the personality measures in medical school admissions, but also would make it totally unacceptable to medical education community.
2. Availability of psychometrically sound measuring instruments: a personality attribute that varies among individuals, varies to some extent, thus can be measured with a psychometrically sound instrument. In particular, in the context of medical education and patient care, the content, construct, criterion-related, and predictive validities; internal consistency (Cronbach’s coefficient alpha) and score stability (test-retest reliability) must be firmly established.
3. Empirical link to clinical competence and patient outcomes: the selection of the pertinent personality attributes must be evidence-based, meaning that convincing empirical evidence must support the significant associations between the selected personality attributes and indicators of clinical competence for doctors-in-training, and patient outcomes for doctors-in-practice. The question is which personality attributes in medical education and patient care can satisfy all of the three aforementioned requirements?

### Is empathy a pertinent personality attribute?

There are some personality attributes that seem germane to clinical competence of doctors-in-training and in-practice. However, as described in a recent review article^3^ at the present time empathy seems to be a unique attribute that can meet all of the three aforementioned requirements.First, there is a consensus that empathy is an essential element of professionalism in medicine.^5^ Thus, it is conceptually relevant to clinical performance and patient outcomes. In the context of medical education and patient care, empathy is conceptualized as “a predominantly cognitive (as opposed to affective or emotional) attribute that involves an understanding (as opposed to feeling) of patients’ pain, experiences, concerns, and perspectives, combined with a capacity to communicate this understanding, and an intention to help.”^6^^-^^8^ This definition makes a distinction between empathy (predominantly a cognitive attribute) and sympathy (predominantly an affective response) which engender different consequences in the context of patient care.^6^^,^^9^Second, there exists a psychometrically sound instrument, the Jefferson Scale of Empathy (JSE), which was specifically developed to measure empathy in the context of medical education and patient care. Prior to the development of the JSE a few instruments had existed for measuring empathy in the general population.^6^ However, none had face and content validities for measuring empathy in medical education and patient care. Relying on the aforementioned definition of empathy, about a decade ago the JSE was developed by our team at Jefferson Medical College to measure empathy in health profession education and patient care.^6^^,^^10^^-^^12^ The JSE contains 20 items, each answered on a 7-point Likert scale, and can be completed in approximately 5-10 minutes.The JSE has gained a broad reputation as a credible research instrument, has been translated into 42 languages, and used in more than 65 countries.^13^ Extensive data have been published in support of the JSE’s psychometric properties (e.g., construct,^10^^-^^12^ criterion-related,^10^^,^^14^^,15^ and predictive validities,^16^ test-retest reliability,^12^ and internal consistency reliability^10^^,^^14^^,15^ on samples of medical students, doctors, and other health profession students and practitioners in the United States^8^^-^^20^ and abroad.^21^^-^^36^ More information about the JSE is posted at: www.jefferson.edu/jmc/crmehc/jse.html.Third, significant empirical associations have been reported between scores of the JSE on the one hand, and measures of clinical competence ^14^ and patient outcomes ^37^^,^^38^ on the other hand. This is the most crucial requirement in support of the credibility of empathy assessment as an additional admission requirement. Despite the large volume of personality research in medical education, the evidence in support of a link between a physician’s personality and patient outcomes is extremely rare.^3^ However, there are some empirical studies that confirm significant associations between JSE scores and faculty’s ratings of students’ clinical competence,^14^ tangible patient outcomes in diabetic patients,^37^^,^^38^ career interest of medical students,^39^ and specialty choice of doctors.^12^

### Persistent hesitation and resistance

Despite the aforementioned evidence, there remains a lingering doubt, persistent skepticism, and a lasting lack of enthusiasm to include assessments of pertinent personality attributes, such as empathy, in medical school admission decisions. Several factors contribute to the hesitation, including an unverified assumption that personality attributes (such as empathy) can be inferred from admission interview, letter of recommendation, personal statements, letter of intent, and essay. Ample evidence suggests that the validity of this assumption remains untested at the present time.^3^^, ^^40^^-^^45^ Some may be also concerned about possibility of respondent’s faking in self-reported personality tests. In response to this concern, we have described approaches to minimize so called “social desirability” response set.^3^^, ^^6^Another reason for the lingering doubt is that the validity evidence for personality measures in medical education is not strong enough to warrant their application in medical school admission decisions. On the surface, this argument appears to make sense; however, the predictive validity coefficients in medical education are often moderate, hovering around 0.30.^3^^,^^42^ Results of some meta-analytic studies show that the predictive validity coefficients of personality tests in general hover around 0.20.^46^^,^^47^ Therefore, the modest validity coefficients are not unique to medical education research and should not deter us from considering the assessment of applicants’ empathy in medical school admission decisions.In addition, some may be concerned about sociopolitical implications for using personality assessment (such as empathy), in medical school admission decisions. They argue that it would deny the opportunity to pursue a medical career for those who are academically qualified based on their scores of the admission tests (e.g., MCAT in North America, and UKCAT in the UK). I would challenge these skeptics to provide empirical evidence that the scores of these admission tests can predict students’ clinical competence and patient outcomes as well as those of the JSE!Medical schools may be willing to buy into the use of empathy assessments for monitoring professional development purposes.^3^ However, it is not sufficient to endorse the use of empathy assessments only for training purposes. It would be more desirable and cost-effective to select applicants who have already developed empathic orientation, than others who are steps behind (by their low scores on the JSE); thus need additional training to enhance their empathic understanding.

### What should be done?

Medical schools are socially accountable to select “qualified” applicants with the best potential to become “good physicians”,^4^^,^^48^ not just those who can successfully pass examinations of recalling factual knowledge in the early years of medical school. To offer or deny the opportunity to applicants to pursue medicine is a critical responsibility of medical schools. Inappropriate decisions during the admission stage would be detrimental to the medical profession, harmful to society, and can jeopardize public safety.The notion of social accountability in medical school admissions could lead to a potentially new legal challenge for medical schools (first brought to my attention by Joe Gonnella, MD). Perhaps, not in a distant future, medical schools could be summoned to the court of law for unprofessional conduct of their graduate, and interpersonal incompetence, and malpractice of those who were admitted to the medical school without assessments of their personal qualities, completed medical school curriculum without development of qualities pertinent to patient care, and granted a medical degree to practice a profession mismatched with their personality and character. To avoid such legal challenges, to render more optimal care, to regain reputation of the profession of medicine, and to reclaim compassionate image of doctors, bold actions must be taken to break free from unverified assumptions, unfounded notions, and sociopolitical considerations. What other evidence is needed to take the action at admission stage?

## References

[1] Gonnella JS Hojat M Erdmann JB Veloski JJ. What have we learned, and where do we go from here? Acad Med. 1993;68:s79-s87. 10.1097/00001888-199302000-00036 8431259

[2] Gonnella JS, Hojat M, Erdmann JB, Veloski JJ. The role of resident performance evaluation in board certification. In: Mancall EL, & Bashook PG, editors. Evaluating residents for board certification. Evanston, IL: American Board of Medical Specialties, 1998. p. 3-14.

[3] Hojat M Erdmann JB Gonnella JS. Personality assessments and outcomes in medical education and the practice of medicine: AMEE Guide No. 79. Med Teach. 2013;35:e1267-e1301. 10.3109/0142159X.2013.785654 23614402

[4] Gonnella JS Hojat M. Medical education, social accountability, and patient outcomes (commentary). Med Educ. 2012;46:3-4. 10.1111/j.1365-2923.2011.04157.x 22150187

[5] Veloski J, ., Hojat M. Measuring specific elements of professionalism: empathy, teamwork, and lifelong learning. In: Stern D, editor. Measuring medical professionalism. Oxford: Oxford University Press; 2006. p. 117-45.

[6] Hojat M. Empathy in patient care: antecedents, development, measurement, and outcomes. New York: Springer, 2007.

[7] Hojat M. Ten approaches for enhancing empathy in health and human services cultures. J Health Hum Serv Adm. 2009;31:412-50. 19385420

[8] Hojat M Vergare M Maxwell K Brainard G Herrine SK Isenberg GA Veloski JJ Gonnella JS. The devil is in the third year: a longitudinal study of erosion of empathy in medical school. Acad Med. 2009;84:1182-91. 10.1097/ACM.0b013e3181b17e55 19707055

[9] Hojat M Spandorfer J Louis DZ Gonnella JS. Empathic and sympathetic orientations toward patient care: conceptualization, measurement, and psychometrics. Acad Med. 2011;86:989-95. 10.1097/ACM.0b013e31822203d8 21694570

[10] Hojat M Mangione S Nasca TJ Cohen MJM. Gonnella JS Erdmann JB Veloski JJ Magee M. The Jefferson Scale of Physician Empathy: development and preliminary psychometric data. Educational and Psychological Measurement. 2002; 61:349-65. 10.1177/00131640121971158

[11] Hojat M Gonnella JS Nasca TJ Mangione S Veloski JJ Magee M. The Jefferson scale of physician empathy: further psychometric data and differences by gender and specialty at item level. Acad Med. 2002;77:s58-s60. 10.1097/00001888-200210001-00019 12377706

[12] Hojat M Gonnella JS Nasca TJ Mangione S Vergare M Magee M. Physician empathy: definition, components, measurement, and relationship to gender and specialty. Am J Psychiaty. 2002;159:1563-69. 10.1176/appi.ajp.159.9.1563 12202278

[13] Hojat M Axelrod D Spandorfer J Mangione S. Enhancing and sustaining empathy in medical students. Med Teach. 2013;35:996-1001. 10.3109/0142159X.2013.802300 23758178

[14] Hojat M Gonnella JS Mangione S Nasca TJ Veloski JJ Erdmann JB Empathy in medical students as related to academic performance, clinical competence, and gender. Med Educ. 2002;36:522-27. 10.1046/j.1365-2923.2002.01234.x 12047665

[16] Hojat M Mangione S Nasca TJ Gonnella JS. Empathy scores in medical school and ratings of empathic behavior 3 years later. J Soc Psychol. 2005;14:663-72. 10.3200/SOCP.145.6.663-672 16334513

[17] Fjortoft N Van Winkle LJ Hojat M. Measuring empathy in pharmacy students. Am J Pharm Educ. 2011;75(6). 10.5688/ajpe756109 21931447 PMC3175671

[18] Fields SK Mahan P Hojat M Tillman P Maxwell K. Measuring empathy in healthcare profession students using the Jefferson Scale of Physician Empathy: health Provider-Student Version. J Interprof Care. 2011;25:287-93. 10.3109/13561820.2011.566648 21554061

[19] Ward J Schaal M Sullivan J Bowen ME Erdmann JB Hojat M. Reliability and validity of the Jefferson Scale of Empathy in undergraduate nursing students. J Nurs Meas. 2009;17:73-88. 10.1891/1061-3749.17.1.73 19902660

[20] Sherman JJ Cramer A. Measurement of changes in empathy during dental school. J Dent Educ. 2005;69:338-45. 15749944

[21] Alcorta-Garza A Gonzales-Guerrero JF Tavitas-Herrera SE Rodrigues-Lara F J Hojat M. Validation of the Jefferson Scale of Physician Empathy in Mexican medical students. Salud Mental. 2005;28:57-63

[22] Di Lillo M Cicchetti A Lo Scalzo A Taroni F Hojat M. The Jefferson Scale of Physician Empathy: preliminary psychometrics and group comparisons in Italian physicians. Acad Med. 2009;84:1198-1202. 10.1097/ACM.0b013e3181b17b3f 19707057

[23] Hsiao CY Tsai YF Kao YC. Psychometric properties of a Chinese version of the Jefferson Scale of empathy- health profession students. J Psychiatr Ment Health Nurs. 2013;20:866-73. 10.1111/jpm.12024 23205565

[24] Kataoka H Koide N Ochi K Hojat M Gonnella JS. Measurement of empathy among Japanese medical students: psychometrics and score differences by gender and level of medical education. Acad Med. 2009;84:1192-97. 10.1097/ACM.0b013e3181b180d4 19707056

[25] Magalhaes E Salgueira AP Costa P Costa MJ. Empathy in senior year and first year medical students: A cross-sectional study. BMC Med Educ. 2011;11:52. 10.1186/1472-6920-11-52 21801365 PMC3163625

[26] Paro H Daud-Gallotti RM Tiberio I C Pinto RMC Martins MA. Brazilian version of the Jefferson Scale of Empathy: Psychometric properties and factor analysis. BMC Med Educ. 2012;12:73. 10.1186/1472-6920-12-73 22873730 PMC3528616

[27] Preusche I Wagner-Menghin M. Rising to the challenge: cross-cultural adaptation and psychometric evaluation of the adapted German version of the Jefferson Scale of Physician Empathy for students (JSPE-S). Adv Health Sci Educ Theory Pract. 2013;18:573-85. 10.1007/s10459-012-9393-9 22923100

[28] Rahimi-Madiseh M Tavakol M Dennick R Naseri J. Empathy in Iranian medical students: a preliminary psychometric analysis and differences by gender and year of medical school. Med Teach. 2010;32:e471-e478. 10.3109/0142159X.2010.509419 21039088

[29] Roh MS. Hahm BJ Lee DH Suh DH. Evaluation of empathy among Korean medical students: a cross-sectional study using the Korean version of the Jefferson Scale of Physician Empathy. Teach Learn Med. 2010;22:167-71. 10.1080/10401334.2010.488191 20563934

[30] Shariat SV Eshtad E Ansari S. Empathy and its correlates in Iranian physicians: a preliminary psychometric study of the Jefferson Scale of Physician Empathy. Med Teach. 2010;32:e417-e442. 10.3109/0142159X.2010.498488 20854147

[31] Shariat SV Habibi M. Empathy in Iranian medical students: measurement model of the Jefferson Scale of Empathy. Med Teach. 2013; 35:e913-e918. 10.3109/0142159X.2012.714881 22938682

[32] Suh DH Hong JS Lee DH Gonnella JS Hojat M. The Jefferson Scale of Physician Empathy: a preliminary psychometric study and group comparisons in Korean physicians. Med Teach. 2012;34:e464-e468. 10.3109/0142159X.2012.668632 22435916

[33] Tavakol S Dennick R Tavakol M. Psychometric properties and confirmatory factor analysis of the Jefferson Scale of Physician Empathy. BMC Med Educ. 2011;11:54. 10.1186/1472-6920-11-54 21810268 PMC3167872

[34] Vallabh K. Psychometrics of the student version of the Jefferson Scale of Physician Empathy (JSPE-S) in final-year medical students in Johannesburg in 2008. South African Journal of Bioethics & Law. 2011;4:63-68

[35] Wen D Ma X Li H Xian B. Empathy in Chinese physicians: preliminary psychometrics of the Jefferson Scale of Physician Empathy (JSPE). Med Teach. 2013;35:609-10. 10.3109/0142159X.2013.774338 23464841

[36] Williams B Brown T Boyle M Dousek S. Psychometric testing of the Jefferson Scale of Empathy health profession students’ version with Australian paramedic students. Nurs Health Sci. 2013;15:45-50. 10.1111/j.1442-2018.2012.00719.x 23279312

[37] Hojat M Louis DZ Markham FW Wender R Rabinowitz C Gonnella JS. Physicians’ empathy and clinical outcomes in diabetic patients. Acad Med. 2011;86:359-64. 10.1097/ACM.0b013e3182086fe1 21248604

[38] Del Canale S Louis DZ Maio V Wang X Rossi G Hojat M Gonnella JS. Physicians’ empathy and disease complications: an empirical study of primary care physicians and their diabetic patients in Parma, Italy. Acad Med. 2012;87:1243-49. 10.1097/ACM.0b013e3182628fbf 22836852

[39] Hojat M Zuckerman M Gonnella JS Mangione S Nasca TJ Vergare M Magee M. Empathy in medical students as related to specialty interest, personality, and perceptions of mother and father. Personality and Individual Differences. 2005;39:1205-15. 10.1016/j.paid.2005.04.007

[40] Walton HJ. Personality assessment of future doctors. J R Soc Med. 1987;80:27-30. 3560123 10.1177/014107688708000112 PMC1290629

[41] Musson DM. Personality and medical education. Med Educ. 2009;43:395-97. 10.1111/j.1365-2923.2009.03358.x 19422485

[42] Ferguson E James D Madeley I. Factors associated with success in medical school: systematic review of the literature. BMJ. 2002;324:952–57. 10.1136/bmj.324.7343.952 11964342 PMC102330

[43] Gelmann EP Stewart JP. Faculty and students as admissions interviewers: Results of a questionnaire given to applicants. J Med Educ. 1975;50:626-28. 1133833

[44] Elam CL Johnson MMS. An analysis of admission committee voting patterns. Acad Med. 1992;67:s72-s75. 9347745

[45] Eddins-Folensbee FF Harris TB Miller-Wasik K Thompson B. Students versus faculty members as admission interviewers: comparisons of ratings data and admission decisions. Acad Med; 2012;87:458-62. 10.1097/ACM.0b013e318249687d 22361799

[46] Ghiselli EE Barthol RP. The validity of personality inventories in the selection of employees. Journal of Applied Psychology. 1953;37:18-20. 10.1037/h0059438

[47] Schmitt N Gooding RZ Noe RA Kirsch M. Meta-analyses of validity studies published between 1964 and 1982 and the investigation of study characteristics. Personnel Psychology. 1984;37:407-21. 10.1111/j.1744-6570.1984.tb00519.x

[48] Gonnella JS Hojat M. Biotechnology and ethics in medical education of the new millennium: physician roles and responsibilities. Med Teach. 2001;23:371-77. 10.1080/01421590120042982 12098384

